# Cultural evolution of emotional expression in 50 years of song lyrics

**DOI:** 10.1017/ehs.2019.11

**Published:** 2019-11-07

**Authors:** Charlotte O. Brand, Alberto Acerbi, Alex Mesoudi

**Affiliations:** 1 Human Behaviour and Cultural Evolution Group, Biosciences, College of Life and Environmental Sciences, University of Exeter, Penryn, UK; 2 Faculty of Science, Department for Early Prehistory and Quaternary Ecology, University of Tübingen, Germany

**Keywords:** cultural evolution, cultural transmission, popular music, sentiment analysis, transmission biases

## Abstract

Popular music offers a rich source of data that provides insights into long-term cultural evolutionary dynamics. One major trend in popular music, as well as other cultural products such as literary fiction, is an increase over time in negatively valenced emotional content, and a decrease in positively valenced emotional content. Here we use two large datasets containing lyrics from *n* = 4913 and *n* = 159,015 pop songs respectively and spanning 1965–2015, to test whether cultural transmission biases derived from the cultural evolution literature can explain this trend towards emotional negativity. We find some evidence of content bias (negative lyrics do better in the charts), prestige bias (best-selling artists are copied) and success bias (best-selling songs are copied) in the proliferation of negative lyrics. However, the effects of prestige and success bias largely disappear when unbiased transmission is included in the models, which assumes that the occurrence of negative lyrics is predicted by their past frequency. We conclude that the proliferation of negative song lyrics may be explained partly by content bias, and partly by undirected, unbiased cultural transmission.

## Graphical abstract

**Figure:**
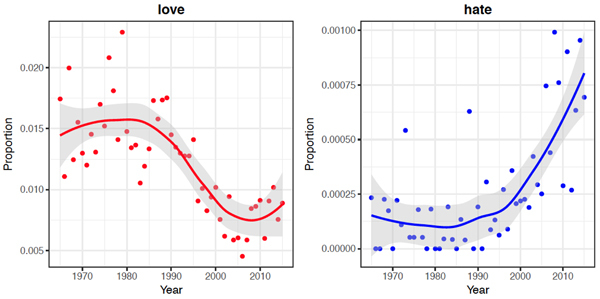

**Media summary:** Increase in negative song lyrics and decrease in positive in 50 years of pop music probably due to cultural drift.

## Introduction

Are the lyrics of contemporary pop songs happier or sadder than the lyrics of the popular songs of previous generations? Do current songs mention love more or less than they used to? Are the pop charts angrier now than in the past, or have they mellowed over time? Millions of people buy and listen to popular music every day (by ‘popular music’ or ‘pop music’, we mean music with wide appeal that is typically distributed to large audiences through the music industry, and not just the specific ‘pop’ genre), and their song choices offer a window into their emotional states and psychological preferences. Changing trends in pop music over time may therefore offer a means of measuring large-scale societal changes. In recent years, the availability of large datasets in electronic format has allowed long-term, population-level cultural dynamics to be identified in an increasingly precise, quantitative way (Michel *et al.* 2011). This, in turn, permits researchers to test hypotheses about cultural trends that previously could only be assessed informally by focusing on a small number of (potentially cherry-picked) cases.

A fruitful area of investigation concerns the analysis of emotions in human cultural expressions. Several tools have been developed to extract the emotional content of texts, known as ‘sentiment analysis’. Some of these provide a classification of how words score on ‘positive’ and ‘negative’ content (Baccianella *et al*. 2010), and others provide additional scores for specific emotions (e.g. how ‘angry’ or ‘sad’ is a text; Pennebaker *et al*. 2007). In most cases, sentiment analysis has been applied on a short-term time scale, such as social media interactions (Lansdall-Welfare *et al.* 2012). However, some researchers have explored a longer time scale, analysing the expression of emotions in several decades of song lyrics (Dodds and Danforth 2010), newspaper articles (Iliev *et al*. 2016), in Grimm's folktales (Mohammad 2013) or in centuries of literary works (Acerbi *et al*. 2013).

The quantitative description of trends is fundamental, but a further necessary step is to understand what drives these trends. Cultural evolution theory (Boyd and Richerson, 1985; Cavalli-Sforza and Feldman, 1981; Mesoudi 2011) provides a series of concepts that allows such an endeavour. Drawing a parallel with genetic evolution, this field focuses on how cultural variation is transmitted from person to person via social learning processes such as imitation, and the processes that change that transmitted variation over time. In particular, cultural evolutionists have focused on transmission or learning biases as key drivers of cultural evolutionary dynamics (R. L. Kendal *et al.* 2018; Rendell *et al.* 2010). Transmission biases are heuristics that individuals use to decide what, when and from whom to copy. They are rule-of-thumb principles such as ‘copy the majority’, ‘copy the successful’ or ‘copy the prestigious’ that allow individuals to adaptively learn from others (Laland 2004). Importantly, different transmission biases give rise to different population-level cultural dynamics. A cultural trait introduced in a population in which, for example, individuals copy mostly from their parents will spread slower than the same cultural trait introduced in a population in which individuals copy mostly from a few successful or prestigious individuals (Cavalli-Sforza and Feldman, 1981).

‘Model-based’ biases describe from whom people learn: for example, a success bias describes a tendency to learn from successful others, and a prestige bias a tendency to learn from prestigious (high status, respected) others. ‘Content-based’ biases describe what kind of information people learn best, owing to its salience or memorability. For example, a bias to transmit emotionally salient content, or negative content, has been found in several laboratory experiments (Bebbington *et al.* 2017; Fessler *et al.* 2014; Stubbersfield *et al.* 2015, 2017). These biases can be compared with unbiased transmission, in which cultural variants are transmitted in equal proportion to their existing frequency in the population. While there are many theoretical models that examine the conditions under which different transmission biases are adaptive and their expected population-level consequences (J. Kendal *et al.* 2009; Rendell *et al.* 2010), and experiments which have tested the predictions of these models in controlled laboratory set-ups (Caldwell and Millen 2008; Mesoudi 2008; Mesoudi and O'Brien 2008; Morgan *et al.* 2012), less research has explored how cultural transmission biases may impact real-life cultural dynamics (although see Acerbi and Bentley 2014; Beheim *et al.* 2014; Miu *et al.* 2018).

In this study, we test the extent to which transmission biases can explain trends in the emotional content of two datasets of English language song lyrics. The first dataset (‘billboard’) contains the lyrics of the songs included in the annual Billboard Hot 100 from 1965 to 2015, a widely known US chart (*n* = 4913 songs). The second dataset (‘mxm’) contains the lyrics of the English language songs present in the musixmatch.com website, the world's largest lyrics platform where users can search and share lyrics, from 1965 to 2010 (*n* = 159,015 songs).

**Figure 1.**
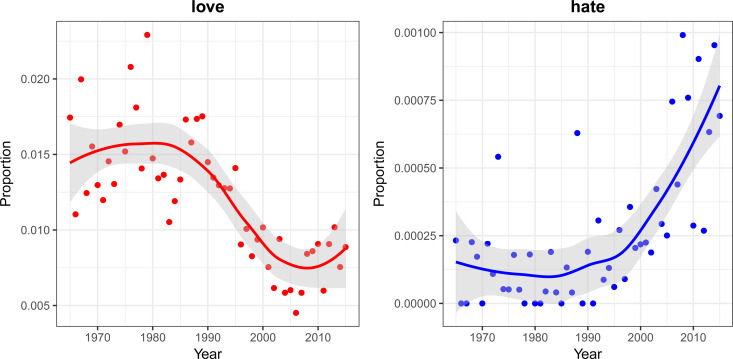
Proportion of the term *‘*love*’* (left panel) and *‘*hate*’* (right panel) in all song lyrics by year for the dataset billboard which contains the lyrics of the songs included in the annual US Billboard Hot 100 (n = 4913 songs). The proportions here are small as we are reporting the proportion of the word out of the total number of words in 100 songs each year (on average 30,000 words, i.e. 300 words/song) and on different scales (the frequency of positive emotion words is usually higher than the frequency of negative emotion words). To have an intuitive idea of the change, from 1965 to 1990, in the top-100 billboard songs, the word *‘*hate*’* was used each year around four or five times overall (30,000*0.00015), whereas now the average is around 24 (30,000 × 0.0008).

Preliminary analyses found a substantial decrease in the use of *positive* emotion-related words coupled with an increase in the use of *negative* emotion-related words in both datasets. Specific emotion-related words show considerable change in use during the time frame considered. For example, use of the term ‘love’ more than halved in both datasets, whereas the term ‘hate’ increased in frequency substantially (see Figure 1 for the ‘billboard’ dataset. The trends are qualitatively similar in the mxm dataset). These results are broadly consistent with previous analyses of song lyrics (DeWall *et al.* 2011; Dodds and Danforth 2010) and literary fiction (Morin and Acerbi 2017), suggesting a general cultural or artistic trend for increasingly negatively valenced emotional expressions.

The main goal of this study is to test hypotheses about possible drivers of these two trends. Before testing for the aforementioned transmission biases, we first checked whether linguistic effects could explain the patterns. We considered (a) a possible increase in slang words, (b) an asymmetric semantic change (for which words denoting negative emotions had acquired positive or neutral connotation, e.g. ‘wicked’, but not vice versa) and (c) a general increase in lyric complexity (although note the latter would predict that both negative- and positive-emotion words would decrease in frequency). After finding that the trend persisted after controlling for these linguistic effects (see Supplementary Material), we then examined whether cultural transmission biases might explain the patterns.

We considered, in a fully preregistered study, three hypotheses derived from cultural evolution theory as outlined above:

(H1) Success bias: the emotional trends result from artists copying the best-selling songs from the preceding years.

(H2) Prestige bias: the emotional trends result from artists copying the songs of ‘prestigious’ artists (independently of the success of the songs) from the preceding years.

(H3) Content bias: there is a general psychological preference for lyrics that reflect negative emotions in songs, thus songs with more negative content rank higher in the charts.

Note that we applied H1 and H2 to both positive and negative content, while H3 was applied only to negative content. This was because, although there is ample evidence for a content bias towards negative emotion, as referenced above, we are not aware of any evidence or theory that would predict a content bias for positive emotions.

After running our preregistered analyses to test these three preregistered hypotheses and depositing a preprint, we received the useful suggestion to also include an additional variable to control for unbiased transmission. Including this additional variable affected the interpretation of our results. Hence below we report both the preregistered analyses without unbiased transmission and the revised analyses including unbiased transmission. The use of unbiased transmission here assumes that the emotional trends of lyrics result from artists randomly copying the lyrics of any of the available songs in the preceding years, without taking into account chart rank or artist prestige.

Our results are mixed. We found a small effect of success bias on the likelihood of a word being positive in the billboard dataset, as well as a small effect of both prestige and content bias on the likelihood of a word being negative in the billboard dataset. However, these effects vastly reduced or became non-existent in the mxm dataset. Moreover, when controlling for unbiased transmission, the effects of success and prestige largely disappeared in both datasets. We therefore conclude that content bias may play a role in the likelihood of using negative emotion words in song lyrics, but that success and prestige biases (as we have implemented them) are not strong enough to explain the trends compared with unbiased transmission. We discuss possible explanations for the apparent content bias effect, as well as our interpretation and implementation of success, prestige and unbiased transmission in relation to the content of song lyrics.

## Methods

### Data preparation

#### Dataset ‘mxm’

We downloaded (in August 2017) the bag-of-words data (*n* = 237,662 songs) from https://labrosa.ee.columbia.edu/millionsong/musixmatch. We used the mxm track ID to retrieve from musixmatch.com the name of the artist(s), the year of first release and the genre for each song.

From this dataset we eliminated songs for which it was not possible to retrieve the year or the artist, as well as songs that did not appear to be in English. To assess if a song was in English we checked whether the word ‘the’ was present at least once. The new dataset contained *n* = 163,551 songs. We additionally filtered the dataset by retaining only the years for which more than 500 songs were present, thus obtaining our final sample of *n* = 159,015 songs, ranging from 1965 to 2010, both included.

Artists’ names were further processed using the cluster function in R library *refinr* (Muir 2018) to cluster and merge similar names (e.g. ‘madonna’, ‘Madonna’, ‘MADONNA’). To disambiguate collaborations we looked for standard separators in artist names (e.g. ‘featuring’, ‘feat.’, ‘feat’, ‘and’, ‘AND’, ‘and’, ‘with’, ‘,’). We considered artists where no separators were found as single artists. We then ran the strings where separators were found (e.g. X and Y) through the list of single artists. If both X and Y were found in this list, then X and Y were considered as single artists occasionally collaborating (e.g. Eminem and Dr. Dre), if not, they were considered a stable collaboration, and a single data point in our models (e.g. Simon and Garfunkel).

#### Dataset ‘billboard’

We downloaded (in May 2017) the data from https://github.com/walkerkq/musiclyrics (*n* = 4913 songs). As the lyrics in the mxm dataset are stemmed (a common practice in digital text analysis: words are reduced to their stems, roughly analogous to their morphological roots, e.g. ‘happily’, ‘happy’, and ‘happiness’ are all reduced to the stemmed form – ‘happi’), we processed the ‘billboard’ dataset in the same way, re-adapting the script used to process the mxm dataset: https://github.com/tbertinmahieux/MSongsDB/blob/master/Tasks_Demos/Lyrics/lyrics_to_bow.py. Henceforth when we use the term ‘word’ or ‘lyric’ we are referring to the stemmed version.

Artists were further processed in the same way as the mxm dataset. Notice the ‘genre’ entry is not present in this dataset as this information was not provided by the Billboard data source, but, importantly, chart position is, which we coded as ‘rank’. Rank is the song's chart position, from 1 to 100, in the yearly Billboard list, where 1 indicates the best-selling and 100 indicates the least-selling song.

#### Sentiment analysis

We used the ‘positive emotions’ and ‘negative emotions’ categories of the text analysis application Linguistic Inquiry and Word Count (Pennebaker *et al.* 2007). These categories are ‘virtually unchanged’ compared with the most recent (2015) version of LIWC. The words were stemmed as described above, and we analysed the lyrics with *n* = 267 negative emotion stems and *n* = 169 positive emotion stems. Thus each word of each song was classified as positive, negative or neither. As noted in the introduction, the practice of analysing the emotional valence of texts using single words as units is common and prevalent in natural language processing studies (a recent review is Ribeiro *et al.* 2016).

### Data analysis

#### Statistical approach

We used Bayesian, aggregated binomial, multilevel models, to examine the effect of prestige, success and content bias on the likelihood of any given word being either positive or not, and negative or not. We conducted all analyses in R version 3.6.0 using the *Rethinking* package (R Core Team 2019; McElreath 2016). We compared WAICs (‘Widely Applicable Information Criteria’, see McElreath 2016) in a model comparison approach as we stated in our preregistration. WAICs can be thought of as a type of information criteria, such as the better-known AIC, but one that uses the entire posterior distribution for estimation rather than a single point estimate. However, it is important to note that we interpreted the parameter estimates of the full models in all cases, based on advice regarding the instability of WAIC estimates for our time series data. Model parameters were said to have an effect on the model outcome if their 89% credible interval did not cross zero (NB. 89% is the default value in the rethinking package). Priors were chosen to be weakly regularising, in order to control for both under- and overfitting of the model to the data. Owing to the large number of data points that each model processed (each model of the mxm dataset processed roughly 29 million words), and skewed samples within genre and artist categories, we had to reparameterise and adapt the models accordingly to ensure sufficient and appropriate convergence. Trace plots, effective sample sizes and Rhat values were used to check for appropriate model convergence throughout.

Full analysis scripts and data are available at www.github.com/lottybrand22/song_lyrics, and were preregistered at https://osf.io/94ubt/

#### Level of analysis

Each word of a song was coded, according to LIWC, as ‘positive’ (when present in the list of positive stems), ‘negative’ (when present in the list of negative stems) or neither. Thus, we implemented separate models for coding positive and negative lyrics (see Tables 1 and 2). Our models are aggregated binomial models, in that the words are aggregated within songs, but each word of a song is modelled as the binomial probability of being positive (or not). The negative models model the likelihood that each word in a song is negative (or not). This takes into account the fact that each song has a different number of words, and negates any need for averaging over words and songs. Each song is not an independent data point, as the data are clustered on artists, genre and year of release. Thus, we implemented varying effects models, allowing adaptively regularising priors for the intercepts of artist, genre and year of release.

**Table 1.** Details of model comparison results for the billboard dataset models. The model with the lowest WAIC and highest proportion of the WAIC weight from each set is in bold. Please note that we always used the full models for inference, as the WAIC standard errors contain a lot of overlap, see *Results* section for more information

Negative/positive lyrics	Model	Parameters	WAIC	Weight	SE
**+ve**	Null	Artist|1 + Year|1	646946.3	0.12	1676.99
**+ve**	**Success bias**	**Success + Artist|1 + Year|1**	**646942**.**5**	**0**.**79**	**1677**.**00**
**+ve**	Prestige bias	Prestige + Artist|1 + Year|1	646948.8	0.03	1677.01
**+ve**	Full	Success + Prestige + Artist|1 + Year|1	646947.9	0.05	1676.93
**+ve**	Full + unbiased transmission	Success + Prestige + unbiased + Artist|1 + Year|1	646952.2	0.01	1676.96
−ve	Null	Artist|1 + Year|1	359482.2	0.00	1442.19
−ve	Success bias	Success + Artist|1 + Year|1	359488.9	0.00	1442.21
−ve	Prestige bias	Prestige + Artist|1 + Year|1	359493.1	0.00	1442.20
−ve	Content bias	Chart position + Artist|1 + Year|1	359472.2	0.22	1442.17
**−ve**	**Full**	**Success + Prestige + Chart position + Artist|1 + Year|1**	**359466**.**4**	**0**.**77**	**1442**.**21**
**−ve**	Full + unbiased transmission	Success + Prestige + Chart position + unbiased + Artist|1 + Year|1	359479.6	0.01	1442.21

**Table 2.** Details of model comparison results for the mxm dataset models. The model with the lowest WAIC and highest proportion of the WAIC weight from each set is in bold. Please note that we always used the full models for inference, as the WAIC standard errors contain a lot of overlap, see *Results* section for more information

Negative/positive lyrics	Model	Parameters	WAIC	Weight	SE
**+ve**	**Null**	**Artist|1 + Genre|1 + Year|1**	**9481478**	**1**	**6651**.**86**
**+ve**	Success bias	Success + Artist|1 + Genre|1 + Year|1	9481503	0	6651.80
**+ve**	Prestige bias	Prestige + Artist|1 + Genre|1 + Year|1	9481502	0	6651.80
**+ve**	Full	Success + Prestige + Artist|1 + Genre|1 + Year|1	9481518	0	6651.89
**+ve**	Full + unbiased transmission	Success + Prestige + unbiased + Artist|1 + Genre|1 + Year|1	9481500	0	6651.79
**−ve**	**Null**	**Artist|1 + Genre|1 + Year|1**	**6602738**	**1**	**6057**.**61**
−ve	Success	Success + Artist|1 + Genre|1 + Year|1	6602763	0	6057.55
−ve	Prestige	Prestige + Artist|1 + Genre|1 + Year|1	6602754	0	6057.52
−ve	Full	Success + Prestige + Artist|1 + Genre|1 + Year|1	6602787	0	6057.53
**−ve**	Full + unbiased transmission	Success + Prestige + unbiased + Artist|1 + Genre|1 + Year|1	6602767	0	6057.54

#### Hypotheses

See Table 1 for specifications of all models. The success bias models assume that the probability that any given word in a song is positive (or negative) can be predicted by the average number of positive (or negative) words of the top-10 songs in the preceding three years of the billboard list. Thus our variable ‘success’ for both mxm and billboard datasets consisted of the average number of positive (or negative) words from the top-10 songs of the billboard dataset, for the preceding three years of the song of interest.

The prestige bias models assume that the probability that any given word in a song is positive (or negative) can be predicted by the average number of positive (or negative) words of the songs of prestigious artists in the preceding three years. We define ‘prestigious’ artists as those that appeared more than 10 times in the Billboard top-100. This results in 86 ‘prestigious’ artists (less than 4% of the total in the billboard dataset).

The content bias models assume that the probability that a word of a song will be negative is predicted by the chart positions of the song in the billboard charts, which we assume reflects a psychological bias, or preference for, negative content in songs.

A control for unbiased transmission is implemented by including an effect for the average number of positive (or negative) words of all songs in the preceding three years. This assumes that the probability that any given word in a song is positive (or negative) increases as the frequency of positive (or negative) words in the population of recent songs increases.

Note that many of these choices are necessarily subjective (e.g. using the top-10 songs for success bias, or more than 10 appearances in the charts for prestige). We invite readers to explore alternative choices by adapting our analysis scripts for use on the openly released data.

## Results

### Model comparison

Our model comparison results suggested that, although some models hold the majority of the weight for the billboard models, it is important to note that their WAIC values are not very different from one another, and the standard errors of the differences show that there is a lot of uncertainty in the model comparison. Furthermore, model comparison is less robust when modelling time-series data. Therefore, we are reporting the results of the full models that include all of the effects of interest, while noting that the model comparison results should be interpreted with caution. We first report the full models without a parameter for unbiased transmission as was originally preregistered. We then report how the results change when controlling for unbiased transmission.

### Models of positive lyrics using the billboard dataset

**Figure 2.**
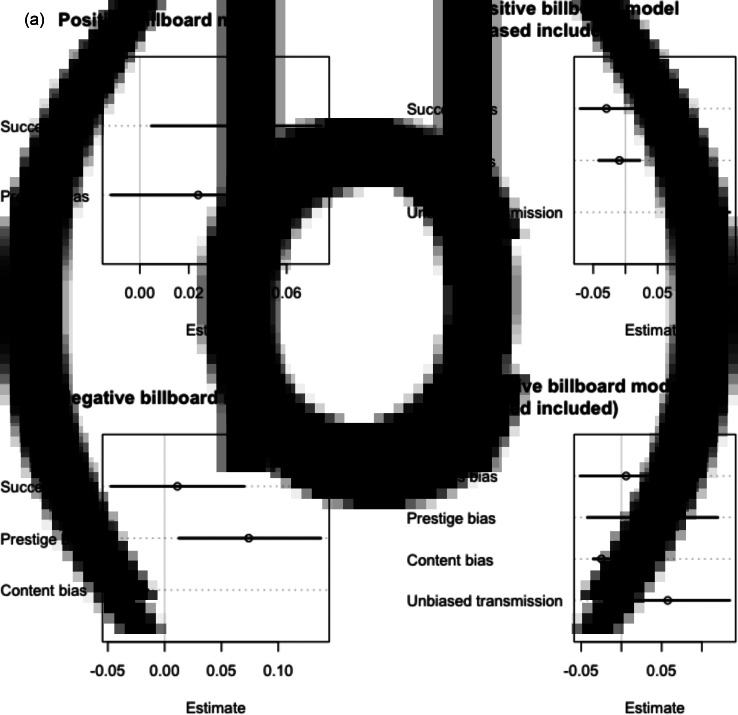
(a) Parameter estimates from the full positive billboard model. Estimates include 89% confidence intervals. Estimates that cross zero are interpreted as not having a strong effect on the probability of a lyric being positive. (b) Parameter estimates from the full positive billboard model with unbiased transmission included. (c) Parameter estimates from the full negative billboard model. (d) Parameter estimates from the full negative billboard mode with unbiased transmission included.

When modelling the likelihood that any word in the lyrics of a song is positive, the full model (Fig. 2a) suggested that success had a small positive effect (mean coefficient estimate: 0.04, 89% confidence interval (CI) [0.00, 0.07], however prestige did not have a detectable effect (0.02, CI [−0.01, 0.06]). These effects are on the log-odds scale, and thus can be interpreted as the odds that a given word is positive increases by 4% as the number of positive words in the previous three years of top-10 songs increases. The model comparison suggests support for H1, suggesting that our hypothesis that success bias contributes to the proportion of positive lyrics was best supported, although there is a lot of uncertainty around the models’ WAIC estimates. However, when controlling for unbiased transmission, the updated full model (Fig. 2b) suggested that neither success nor prestige had a detectable effect (−0.03, CI [−0.07, −0.01], −0.01 CI [−0.04, 0.02]), but that our implementation of unbiased transmission predicted the likelihood of a given word in the lyrics of a song being positive (0.12, CI [0.07, 0.16]).

### Models of negative lyrics using the billboard dataset

When modelling the likelihood that any word in the lyrics of a song is negative, the full model (Fig. 2c) suggested that prestige had a positive effect (mean coefficient estimate: 0.08, 89% CI [0.01, 0.14]; however success did not have a strong effect (0.01, CI [−0.05, 0.07]). Content bias had a negative effect, meaning that songs that had a numerically lower chart position (i.e. closer to the top of the charts at 1 rather than 100) increased the log odds of a lyric being negative (−0.02, CI [−0.04, −0.01]). When controlling for unbiased transmission (Fig. 2d), the model suggested that neither success nor prestige had a detectable effect on the likelihood of a lyric being negative (0.01, CI [−0.05, 0.06], 0.04 CI [−0.04, 0.12]). However content bias retained its effect, in that a lower chart position increased the likelihood of a lyric being negative (−0.02, CI [−0.04, −0.01]). There was not strong evidence of unbiased transmission (0.06, CI [−0.02, 0.13]).

### Models of positive lyrics using the mxm dataset

**Figure 3.**
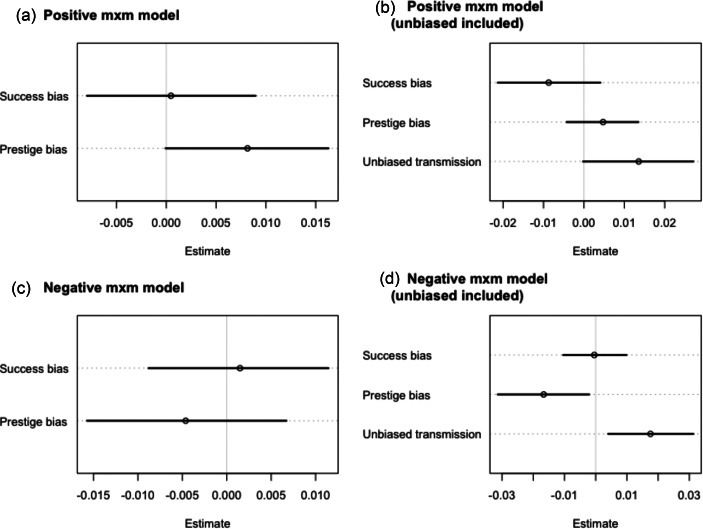
(a) Parameter estimates from the full positive mxm model. Estimates include 89% confidence intervals. Estimates that cross zero are interpreted as not having a strong effect on the probability of a lyric being positive. (b) Parameter estimates from the full positive mxm model with unbiased transmission included. (c) Parameter estimates from the full negative mxm model. (d) Parameter estimates from the full negative mxm model with unbiased transmission included.

When modelling the likelihood that any word in the lyrics of a song in the mxm dataset is positive (Fig. 3a), the full model suggested that prestige had a small positive effect (mean coefficient estimate 0.01, 89% CI [0.00, 0.02]; however success did not have an effect (0.00, CI [−0.01, 0.01]). The prestige effect is much smaller than those from the billboard models. The model comparison suggested that the best model was the null model, suggesting that our parameters for success and prestige did not improve the model fit more than simply including the varying effects for artist, genre and year of release. Furthermore, when controlling for unbiased transmission (Fig. 3b) we found no effect of prestige and success (0.00, CI [0.00, 0.01], −0.01 CI [−0.02, 0.00]) and weak evidence of unbiased transmission (0.01, CI [0.00, 0.03]).

### Models of negative lyrics using the mxm dataset

When modelling the likelihood that any word in the lyrics of a song is negative, the full model (Fig. 3c) suggested that neither prestige nor success had an effect (prestige 0.00, 89% CI [−0.02, 0.01]; success 0.00, CI [−0.01, 0.01]). Furthermore, the model comparison indicated that the best model was the null model, suggesting that our parameters for success and prestige did not improve the model fit more than simply including the varying effects for year, artist, genre. Similarly, when controlling for unbiased transmission (Fig. 3d) we found no effect of prestige and success, (−0.02, CI [−0.03, 0.01], 0.00 CI [−0.01, 0.01]) and weak evidence of unbiased transmission (0.02, CI [0.00, 0.03]).

## Discussion

We analysed the emotional content of song lyrics in over 160,000 songs spanning the years 1965–2015. We found that the frequency of negative words increased over time, whilst the frequency of positive words decreased over time, and asked whether these patterns could be attributed to cultural transmission biases such as success bias, prestige bias, content bias or unbiased transmission. In the billboard dataset, containing top-100 songs from 1965 to 2015, we found an effect of unbiased transmission on positive lyrics, and an effect of content bias on negative lyrics. For the larger mxm databases we only found weak effects of unbiased transmission for both negative and positive lyrics.

The effects we found in all models are extremely small. This is partly because we analysed the data on the scale of each word, negating any need for averaging over lyrics and songs. Thus, the relative increase or decrease in the log odds is understandably small. Furthermore, our implementation of transmission biases is necessarily indirect and simplified given that we lack direct observations of song lyrics being copied. It is therefore unsurprising that the effects vastly reduced or disappeared when controlling for unbiased transmission, given how many other factors must be at play in the generation of song lyrics, both directional biases such as those we explored here and random processes (Bentley *et al.* 2007). For example, prestige can be realised in myriad ways (Jiménez and Mesoudi 2019), particularly in the music industry. The effect of various recording companies, the extent of media attention outside of the charts and the amount of money spent on music promotion may all play a significant role in an artist's apparent prestige, and is not necessarily restricted to the content of their music. Our implementation of ‘prestige’ as predominance in the charts therefore only captures one specific aspect of musical prestige.

The effect of unbiased transmission is, however, the largest and most consistent in all of our models. This result suggests there may be an effect of random drift, or random copying, in the emotional content of song lyrics over time. This is consistent with previous work showing that random copying can explain changes in the popularity of dog breeds, baby names and popular music (Bentley *et al.* 2007; Hahn and Bentley 2003), as well as archaeological pottery and technological patents (Bentley *et al.* 2004). Thus, rather than song-writers being influenced by the most prestigious or successful artists, they may simply be influenced by the emotional content of any of the available song lyrics in the previous timestep, which may happen to increase in negativity or decrease in positivity owing to small fluctuations. As in previous work, our results do not provide evidence of literal random copying by individuals as we do not have direct access to individual's copying decisions. Instead, random drift is posed as a baseline against which to compare evidence of other copying biases. It is possible that the population-wide patterns are not a result of unanimous random copying, but owing to a multitude of idiosyncratic causes that collectively cancel each other out to create the appearance of random copying (Hoppitt and Laland 2013). In this sense, any small fluctuation in negative words owing to a particular historical event, or owing to the emergence of a more negatively biased genre, may have caused an initial increase in negative lyrics, which became exacerbated by random drift.

The presence of a content bias in the likelihood of negative lyrics occurring in the billboard songs is noteworthy. This result suggests that songs with more negative lyrics are more successful in general, perhaps reflecting either a general negativity bias (Bebbington *et al.* 2017; Fessler *et al.* 2014) or an art-specific, or music-specific, negativity bias. Similar trends favouring negative emotions vs positive ones in other artistic domains support our finding. As mentioned above, Dodds and Danforth (2010) documented a decrease in frequency of positively valenced words, and an increase in negatively valenced ones in pop song lyrics (a similar result was found in DeWall *et al.* 2011). Morin and Acerbi (2017) found a similar pattern in centuries of literary fiction, with a general decrease in the frequency of words denoting emotions, explained by a decrease in words denoting positive emotions, whereas the frequency of negative words remained constant. It is worth noting that we were unable to look for content bias (with our implementation) in the mxm data as there was no ranking system. One possible way of determining the popularity or use of a song could be to look at how many times, or how often, its lyrics are searched for, and whether this correlates with negative content.

In general, the idea that negative emotions would be privileged in art is consistent with the hypothesis that artistic expressions may have an adaptive function, in particular as simulation of social interactions (Mar and Oatley 2008). According to this view, developed with literary fiction in mind but potentially generalisable to other expressive forms, art would provide hypothetical scenarios where we can test and train, with no risk, our cognitive and emotional reactions. From this perspective, simulating negative events is more useful than simulating positive ones (Clasen 2017; Gottschall 2012). Art expressing negative emotions, in addition, may hold more value for audiences seeking comfort from the knowledge that others also experience negative emotions. Indeed, studies have shown that people underestimate the prevalence of others’ negative emotions, and this underestimation exacerbates loneliness and decreases life satisfaction (Jordan *et al.* 2011). Furthermore, suppressing rather than reappraising negative emotions decreases self-esteem and increases sadness (Nezlek and Kuppens 2008)(Nezlek & Kuppens, 2008). This hypothesis is worth investigating in future research.

Our varying effects models suggested that most of the variation lay between artists. However, genre also showed considerable variation. We were unable to control for genre in the billboard data as genre information was not available with this dataset. This could provide a partial explanation for the differing results between the billboard and mxm datasets; indeed, Dodds and Danforth (2010) attributed the decrease in emotional valence within pop song lyrics to the emergence of more negative genres such as heavy metal and punk. Future work investigating the variation of emotional expression between different genres of music would be valuable. A further limitation of this study is that we restricted our analysis to comparing the content of each song with that of the songs from the previous three years of songs. Mechanistically this suggests that songs that are currently in the charts influence song-writers who are writing within three years of chart success, assuming that the time it takes to get from the song-writing process to chart success is three years or less. It is possible that these effects are stronger or weaker at different time points, such as within one or five years of chart success. Furthermore, although we controlled for artist, many songs in the billboard charts are in fact written by specially designated song-writers, such as Max Martin.

Overall this research contributes to the growing body of work attempting to quantitatively study trends in the domain of music (Youngblood 2019; Savage 2019; Mauch *et al.* 2015; Ravignani *et al.* 2017). Our starting result of an increase in negative emotions and decrease in positive ones in song lyrics is paired with similar findings regarding acoustic qualities. Using the same Billboard top-100 songs that we analysed, Schellenberg and von Scheve (2012) found an increase in minor mode and a decrease in the average tempo, which indicates that the songs become more sad-sounding through time. This seems to be part of a longer trend in Western classical music, where the use of the minor mode increased over a 150-year period from 1750 to 1900 (Horn and Huron 2015). The relationship between minor tone and negative valence of lyrics has been also studied, and confirmed, quantitatively (Kolchinsky *et al.* 2017). Analogously, studying more than 500,000 songs released in the UK between 1985 and 2015, Interiano *et al.* (2018) found a similar decrease in ‘happiness’ and ‘brightness’, coupled with a slight increase in ‘sadness’ (these high-level features result from algorithms analysing low-level acoustic features, such as the tempo, the tonality, etc.). They also found the puzzling result that, despite a general trend towards sadder songs, the successful hits are, on average, happier than the rest of the songs. In the same way, whereas we found that the higher the position in the billboard chart the more negative a song is, billboard songs are *as a whole* more positive than the songs in the mxm dataset, which contains more (and less successful) songs.

In this study we used cultural evolutionary theory to try to explain patterns in one of the most pervasive of human cultural practices, music production. More specifically, we tried to detect whether any particular transmission bias best explained the changing patterns of emotional expression over time. We conclude that, although we found weak evidence of success and prestige biases, these were overwhelmed by an effect for unbiased transmission. The presence of a content bias for negative lyrics remained, and this may be a contributing factor to the increasing in negative lyrics over time. A potential explanation for these results is that a multitude of transmission biases and other causes are at play. It is likely that small shifts, for example owing to historical events or the emergence of particular genres, may have nudged the production and transmission of negative and positive lyrics in opposite directions, and random copying exacerbated this trajectory. These possibilities should be explored more in future work. Overall, the exercise of precisely analysing large datasets to explain cultural change, if refined on relatively benign cultural trends such as pop music, could eventually be more expertly applied to areas of greater societal importance and impact, such as shifts in political beliefs or moral preferences.

## Supplementary material

For supplementary material accompanying this paper visit http://dx.doi.org/10.1017/ehs.2019.11. click here to view supplementary material
