# Supplementary material for: Cultural evolution of emotional expression in 50 years of song lyrics
Source: Evol Hum Sci. 2019 Nov 7;1:e11. doi: 10.1017/ehs.2019.11 (PMC10427273; doi:10.1017/ehs.2019.11)
Supplement: Supplementary file 1 [file S2513843X19000112sup001.docx]

**Supplementary Information for:**

**Cultural evolution of emotional expression in 50 years of pop song lyrics**

C.O. Brand, A. Acerbi & A. Mesoudi

**General trends**

Our goal is to explain the decrease in positive emotions and the increase in negative emotions in the two datasets “billboard” and “mxm”.

**Figure 1:** Proportion of negative emotions-related words on the total number of words for all songs, by year.

**Figure 2:** Proportion of positive emotions-related words on the total number of words for all songs, by year.

**Linguistic hypotheses**

We first considered three “linguistic” hypotheses, as they assume that the changes in emotional content can be explained by changes in linguistic usage, and they are not directly related to cultural changes. The three hypotheses are the following:

*L1: Increase in slang/swear word usage.* The increase in negative emotions is due to the introduction of slang/swear words.

*L2: Asymmetric semantic changes.* The increase in negative emotions is due to the fact that negative words have switched to positive meaning (e.g. “terrific”) or to be both positive and negative (e.g. “crazy”) more than positive words did;

*L3: Increase in lyrics complexity.* An increase in the general complexity of lyrics explain the decrease in positive emotion words.

Notice that (i) the linguistic hypotheses can account for the increase in negative emotions (L1-L2) or the decrease in positive (L3) but none of them can, taken alone, explain both, and (ii) the fact that linguistic hypotheses are confirmed do not necessarily exclude that “cultural” hypotheses are not. If, for example, L1 is true, i.e. the increase in negative emotions is mainly due to the introduction of slang/swear words, we are still interested in understanding whether songs with negative (i.e. slang/swear) words got widespread because they were more successful, or because prominent artists were using these words.

*L1: Increase in slang/swear word usage*

Slang terms and swear words are more often associated with negative than positive emotions. The increase in slang/swear words usage could be due to several reasons, including, for example, a cultural switch towards more acceptance of swear and taboo words. An alternative explanation, with the same overall effect, is a faster turnover of slang/swear words, due to an example of the “euphemism treadmill”. If everybody starts to use a slang word (e.g. “bugger”) it will lose the severity of its meaning (as people habituate to it) and it will be replaced by another word to convey the original negative meaning. As we use contemporary word-categories to assess the emotional content, this would create a bias for which negative words increase in frequency.

Prediction:

Removing the slang/swear words in the LIWC “negative emotions” category removes the increase in negative emotion words over time.

Analysis:

4 independent raters manually flagged negative words that were also slang/swear words in the LIWC “negative emotions” category. We eliminate the words that were rated as slang/swear words by the majority (at least 3) of the raters, and we analysed the same datasets.

negative_score_bb - slang/swear ~ year

negative_score_mxm - slang/swear ~ year

Results:

The increase in negative emotions words over time is reduced, but it is still present (p=0.0142, Spearman’s rank correlation), for the “billboard” dataset, and remains unchanged for the “mxm” dataset (Figure 1). Figure 2 shows the contribution of the single words (excluding slang/swear words) to the trend.


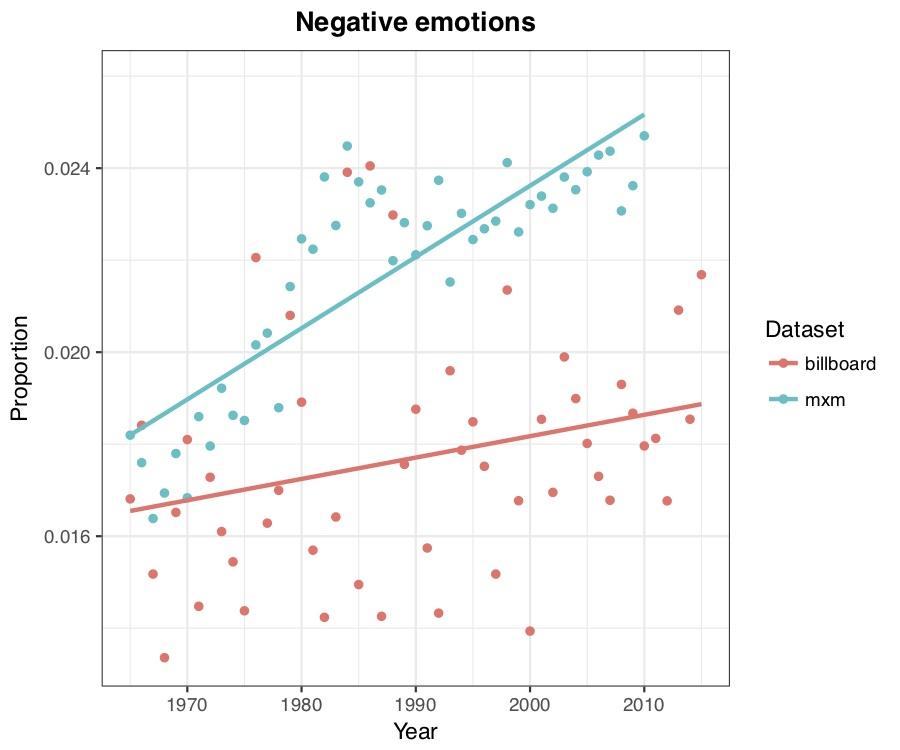


**Figure 3:** Proportion of negative emotions-related words (excluding slang/swear words), on the total number of words for all songs, by year.


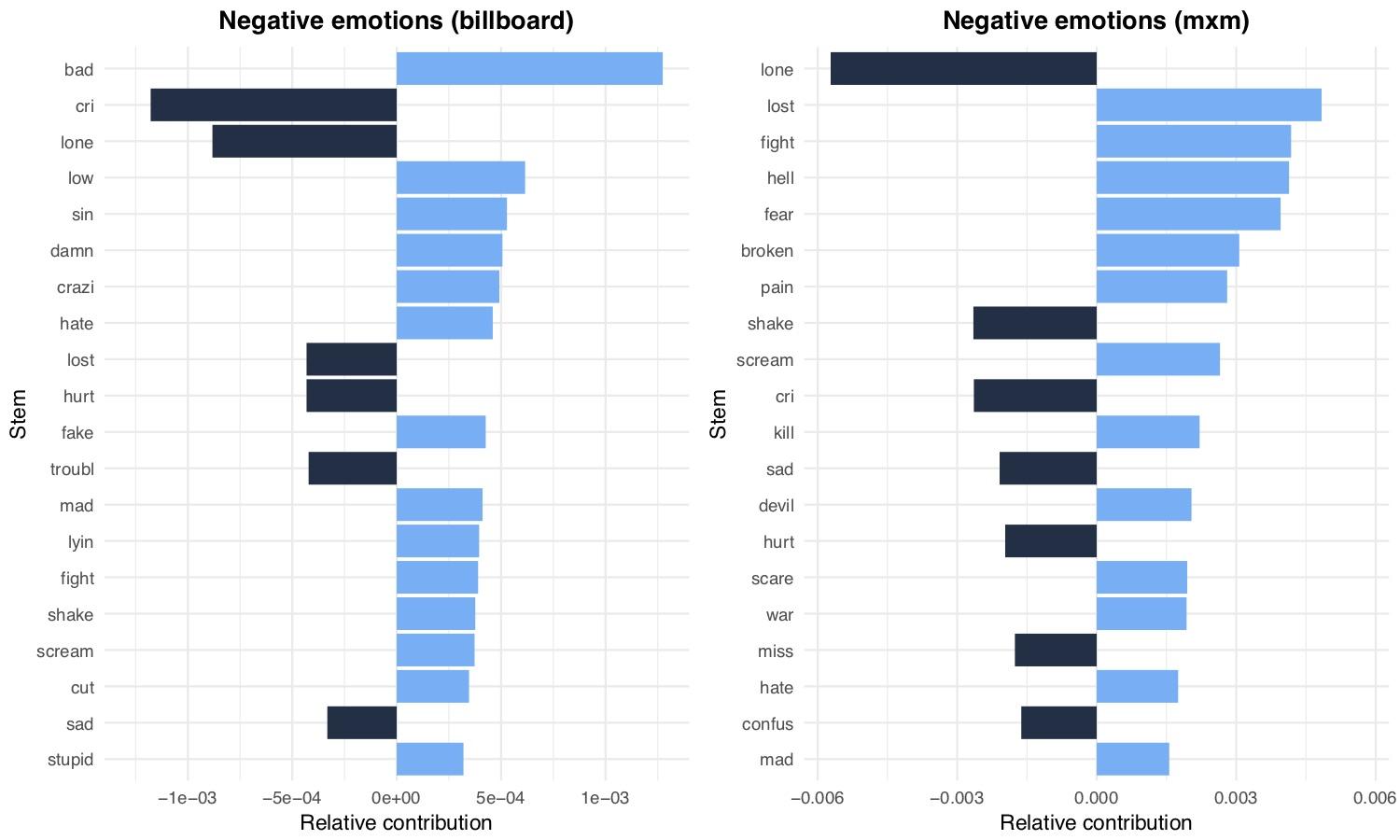


**Figure 4:** Relative contribution of the most contributing 20 negative emotions-related (excluding slang/swear words) stems for the datasets “billboard” and “mxm”.

*L2: Asymmetric semantic changes*

Words denoting negative emotions have acquired through time a positive connotation (e.g. “terrific”) or they are used both in a positive and negative connotation (e.g. “crazy”), while this did not happen - or happened to a lesser extent - for positive emotions. If this is the case, the increase in negative emotions is an indication of this semantic widening and not of a separate cultural/artistic trend.

Prediction:

Contemporary negative words in LIWC show, through the years considered by our analysis, semantic shift towards positive meaning or semantic widening or both. The same does not happen for contemporary positive words in LIWC.

Analysis:

We downloaded data from <https://nlp.stanford.edu/projects/socialsent/> - see in particular “Historical sentiment lexicons for the last 150 years of English (by decade) - Sentiment scores for frequent words”. These data represents estimates of semantic change of the top 5000 non-stop words (i.e. excluding words such as “the”, “is”, “then”, etc.) in each decade from 1850 to 2000 (Hamilton et al., 2016).

For each word, the estimates provide an emotional score (how positive or negative the word is) and the standard deviation (that gives an indication of semantic widening). We considered the words that were present in the LIWC categories for negative and positive emotions that we used for the song lyrics analysis. Starting from the unstemmed version of LIWC we found 175 negative emotions-related words and 237 positive emotions-related words in the *socialsent* dataset. We considered only decades from 1950 to 2000 for the present analysis.

Results:

From a visual inspection, the roles of semantic shift (see figure 3 and 5) and semantic widening (see figure 4 and 6) do not seem important in positive or negative emotions.


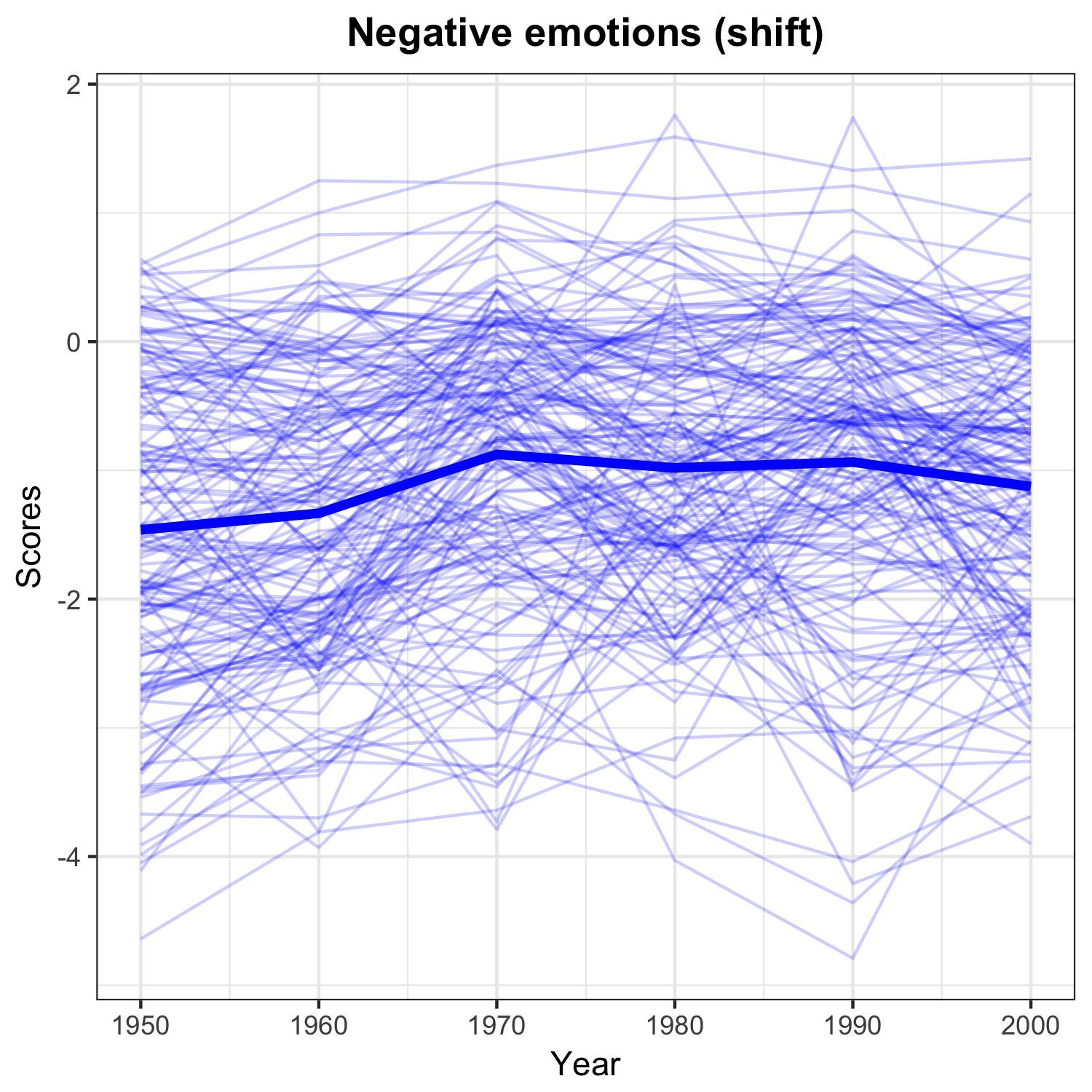


**Figure 5:** Estimated semantic shift for negative emotions.


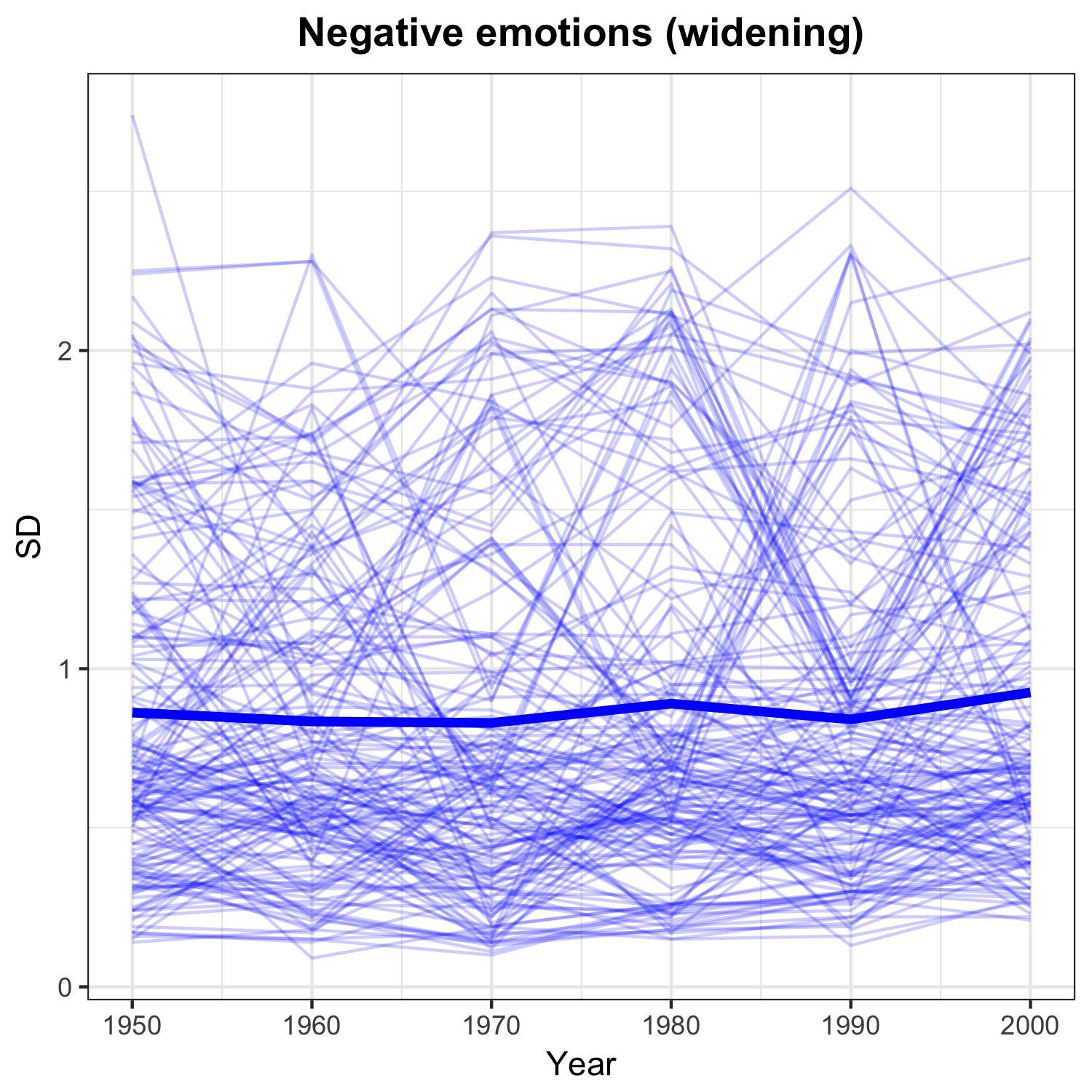


**Figure 6:** Estimated semantic widening for negative emotions.


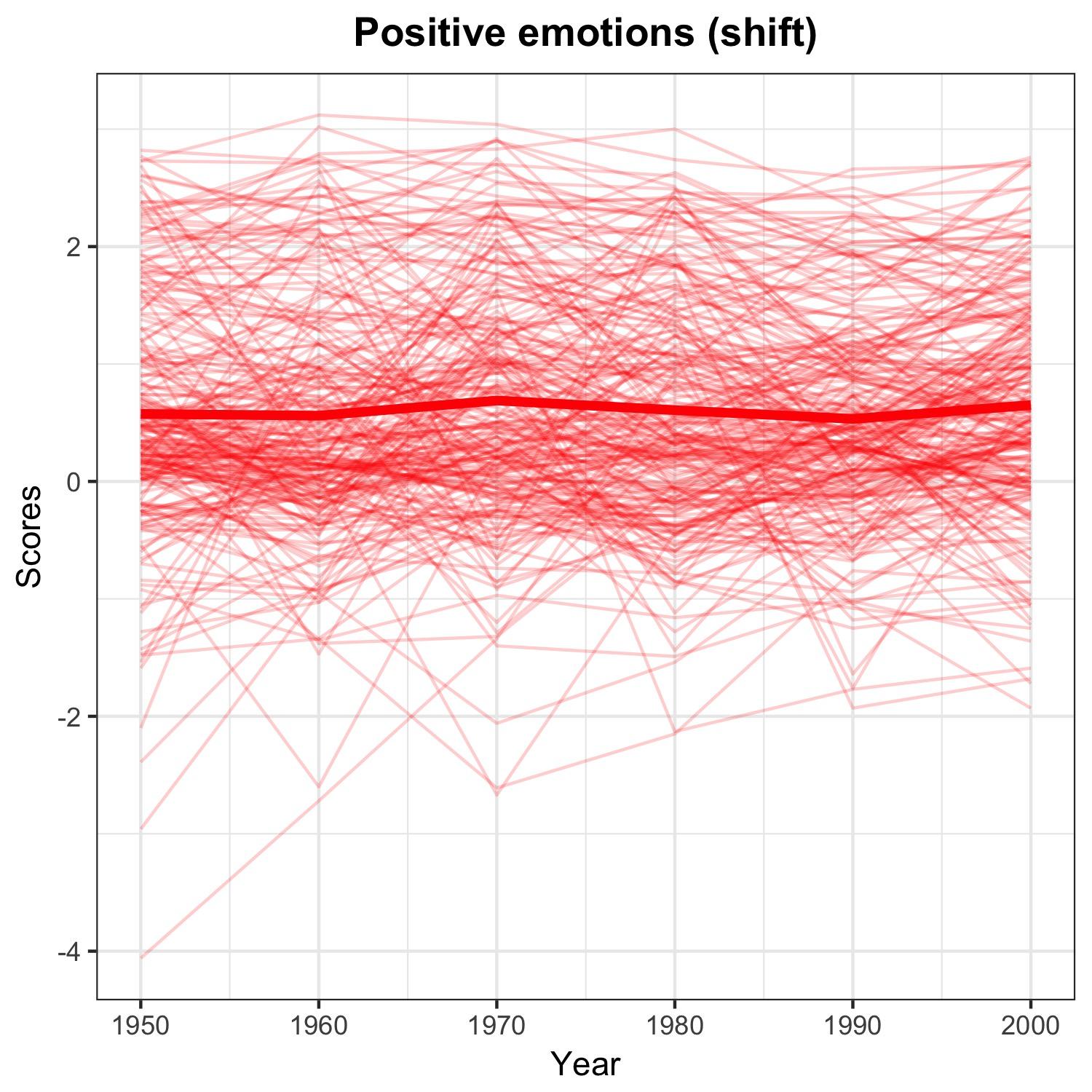


**Figure 7:** Estimated semantic shift for positive emotions.


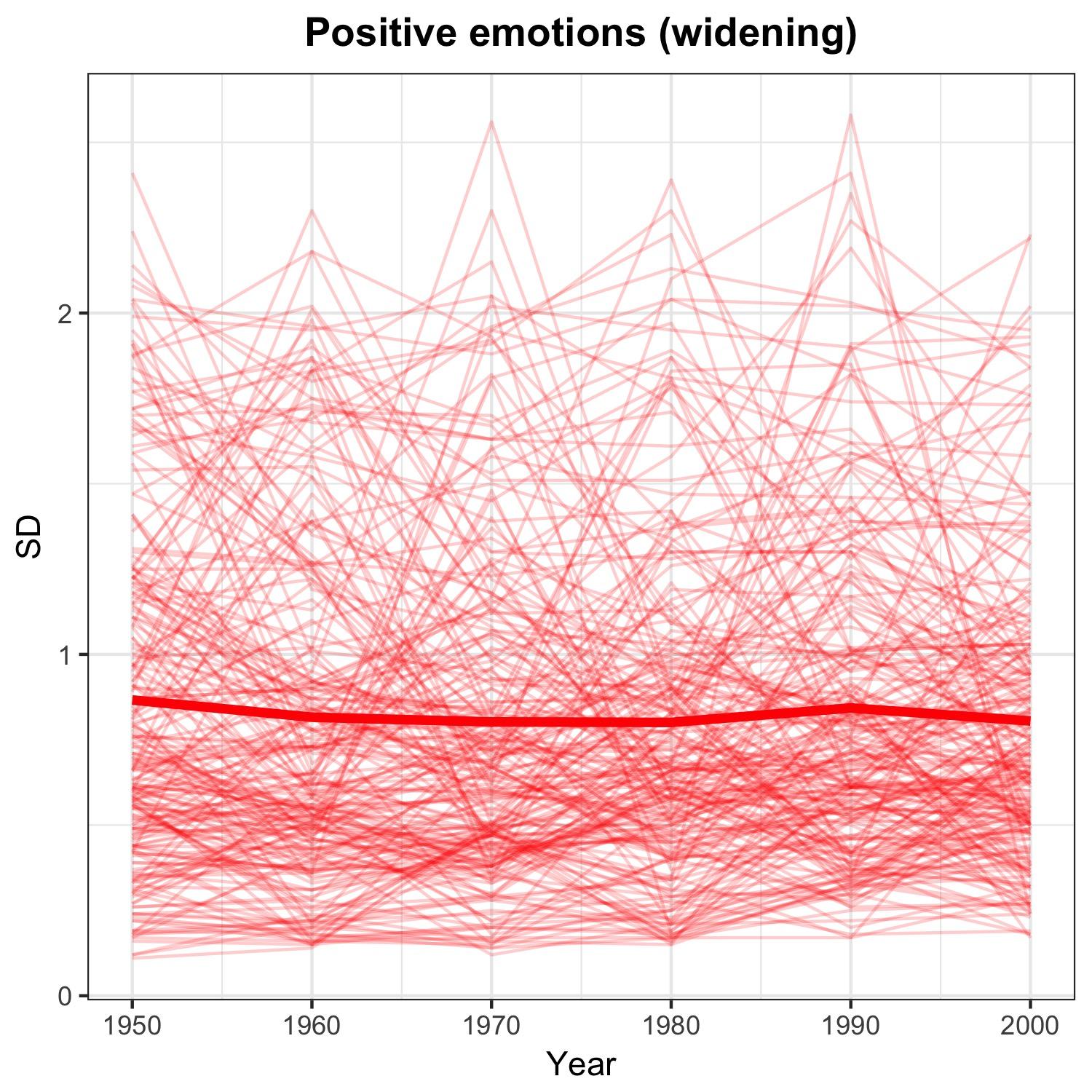


**Figure 8:** Estimated semantic widening for positive emotions.

*L3: Increase in lyrics complexity*

Song lyrics had become more complex through time. We can measure complexity as the total number of words (tokens), the total number of different words in each song (types) and the ratio types/tokens, which measure how repetitive a song is. If song lyrics are becoming less repetitive, *all* words, including positive emotions-related words decrease in frequency.

Prediction:

The ratio types/tokens increases through time

Analysis:

ratio_types/tokens_billboard ~ year

ratio_types/tokens_mxm ~ year

Results:

Figure 7 and Figure 8 show the ratio type/tokens trends through years for the “billboard” and “mxm” dataset respectively. There is no increase.


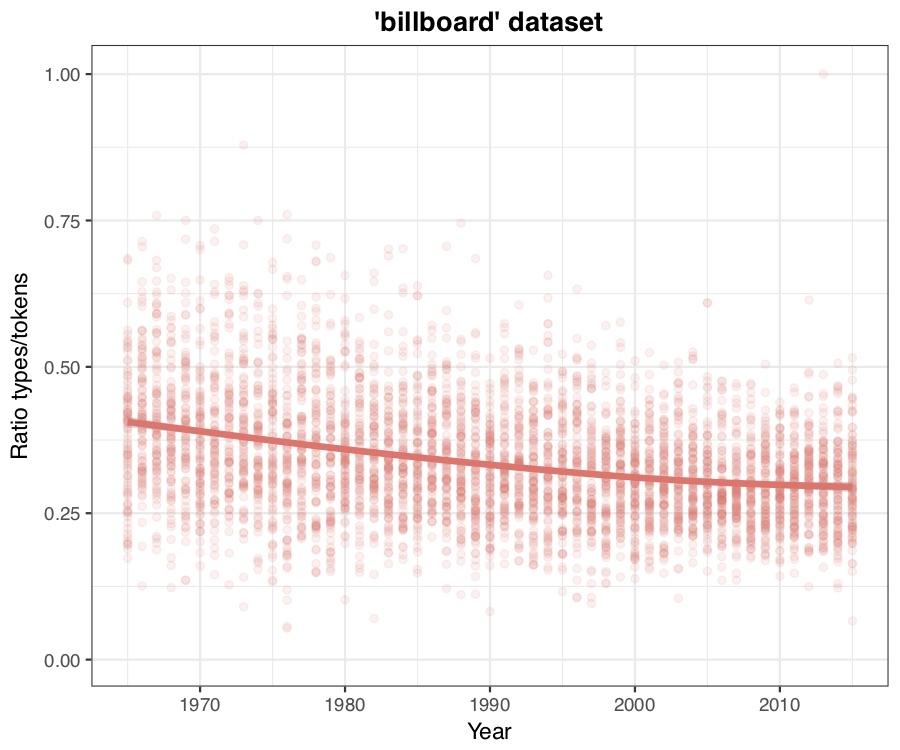


**Figure 9:** Ratio types/tokens through years for the “billboard” dataset. Each point represents a song, the continuous line is the average.


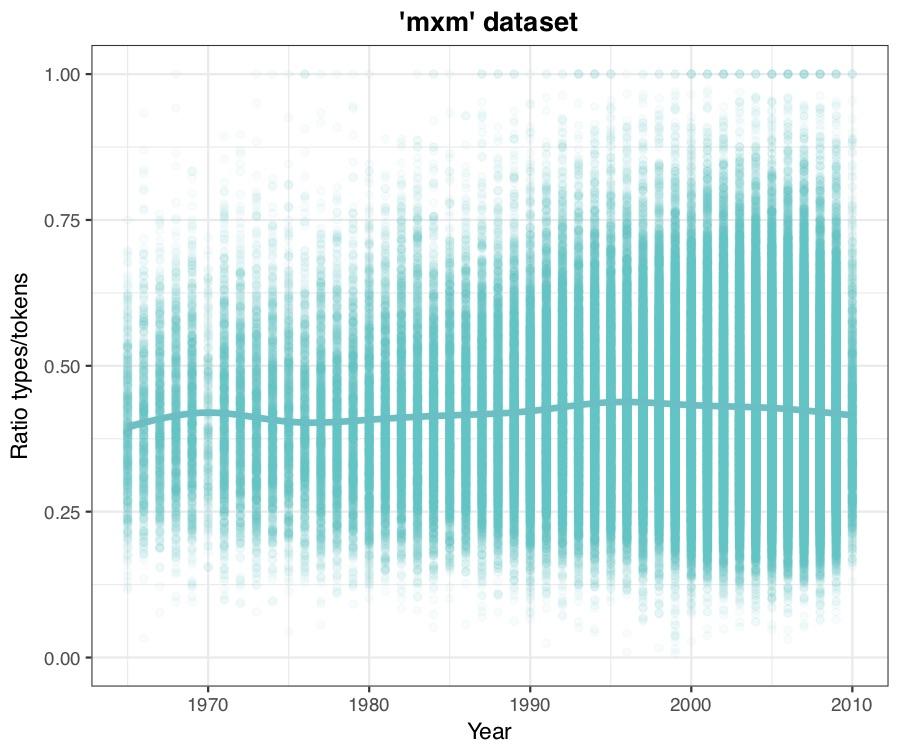


**Figure 10:** Ratio types/tokens through years for the “mxm” dataset. Each point represents a song, the continuous line is the average.

**Variation in year, artist, and genre (where available):**

The following plots display the marginal posterior distributions of the standard deviations of each varying intercept in each of the full models from the main manuscript.


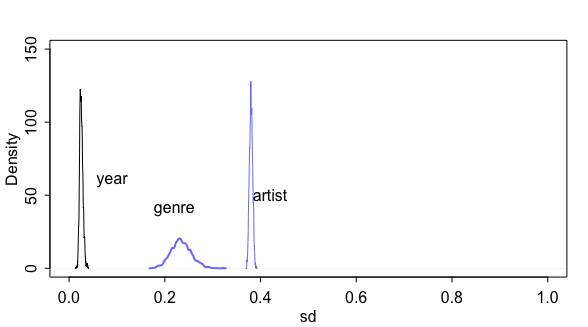


**Figure 11:** Estimates of the standard deviations for year, genre and artist in the full model analysing positive lyrics in the mxm dataset


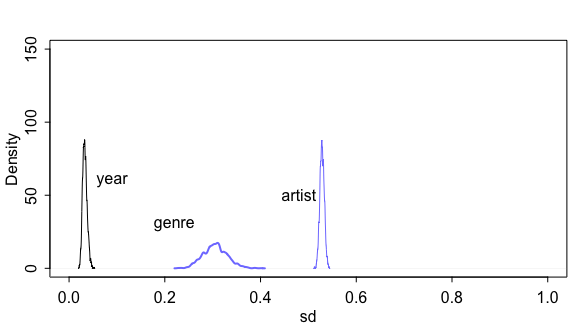


**Figure 12:** Estimates of the standard deviations for year, genre and artist in the full model analysing negative lyrics in the mxm dataset


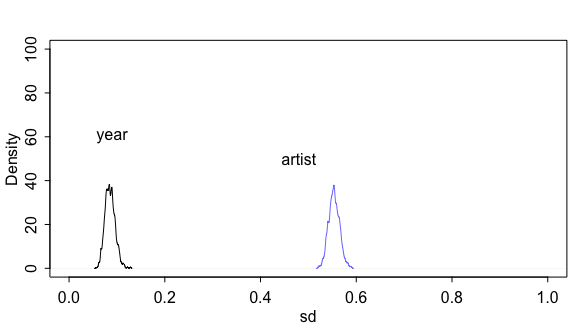


**Figure 13:** Estimates of the standard deviations for year, genre and artist in the full model analysing positive lyrics in the billboard dataset


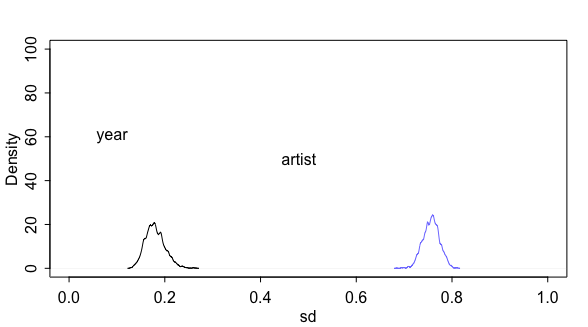


**Figure 14:** Estimates of the standard deviations for year, genre and artist in the full model analysing negative lyrics in the billboard dataset

**References**

Hamilton, W. L., Clark, K., Leskovec, J., & Jurafsky, D. (2016). Inducing domain-specific sentiment lexicons from unlabeled corpora. In *Proceedings of the Conference on Empirical Methods in Natural Language Processing. Conference on Empirical Methods in Natural Language Processing*.
